# Quantum optical signatures in strong-field laser physics: Infrared photon counting in high-order-harmonic generation

**DOI:** 10.1038/srep32821

**Published:** 2016-09-07

**Authors:** I. A. Gonoskov, N. Tsatrafyllis, I. K. Kominis, P. Tzallas

**Affiliations:** 1 Foundation for Research and Technology-Hellas, Institute of Electronic Structure & Laser, P.O. Box 1527, GR-71110 Heraklion (Crete), Greece; 2 Max Planck Institute of Microstructure Physics, Weinberg 2, D-06120 Halle, Germany; 3 Department of Physics, University of Crete, 71103 Heraklion, Greece

## Abstract

We analytically describe the strong-field light-electron interaction using a quantized coherent laser state with arbitrary photon number. We obtain a light-electron wave function which is a closed-form solution of the time-dependent Schrödinger equation (TDSE). This wave function provides information about the quantum optical features of the interaction not accessible by semi-classical theories. With this approach we can reveal the quantum optical properties of high harmonic generation (HHG) process in gases by measuring the photon statistics of the transmitted infrared (IR) laser radiation. This work can lead to novel experiments in high-resolution spectroscopy in extreme-ultraviolet (XUV) and attosecond science without the need to measure the XUV light, while it can pave the way for the development of intense non-classical light sources.

Strong-field physics and attosecond science^1^,^2^,^3^,^4^ have been largely founded on the electron recollision process described by semi-classical approaches^4^ treating the electron quantum-mechanically and the electromagnetic field classically. This is because the high photon number limit pertinent to experiments with intense laser pulses appears to be adequately accounted for by a classically-described electromagnetic wave, which is not affected by the interaction.

In the semi-classical approaches (known as three-step models) used for the discription of the HHG process, the electron tunnels through the strong-laser-field-distorted atomic potential, it accelerates in the continuum under the influence of the laser field and emits XUV radiation upon its recombination with the parent ion. Thus, the motion of the electron in an electromagnetic field is at the core of the recollision process. In the strong-field regime, this motion is well described by non-relativistic semi-classical Volkov wavefunctions, obtained by solving the TDSE for a free electron in a classically-described electromagnetic field.

Extending the semi-classical Volkov wave functions into the quantum-optical region is non-trivial and, to our knowledge, a closed form solution of the quantized TDSE with a coherent-state light field has never been obtained before. Although an accurate calculation of the properties of the XUV radiation emitted from a gas phase medium requires the consideration of the driving IR laser bandwidth and the propagation effects in the atomic medium, the fundamental properties of the interaction can be adequately explored with the single-color single-atom interaction, as has been done in the pioneering work of Lewenstein *et al*.^4^. In this work we develop a quantized-field approach for an ionized electron interacting with light field in a coherent state. We obtain a closed-form solution of the TDSE, which contains complete information about the laser-electron quantum dynamics during the interaction, and use it to describe the HHG process. Differently than previous approaches^5^,^6^,^7^,^8^, we describe the XUV emission as far-field dipole radiation by using an initially coherent laser state and the obtained closed-form electron-laser wave function, named “quantum-optical Volkov wave function”. Our approach consistently extends the well-known semi-classical theories^4^, since from the obtained quantum-optical wave function we can retrieve the semi-classical Volkov wave functions by averaging over the light states. This is of advantage, since all the results of the semi-classical theory (like harmonic spectrum, electron paths, ionization times, recombination times, etc.) can be retrieved from- and utilized in our quantized-field approach.

Going beyond the reach of the semi-classical approach, we find that the quantum-optical properties of the HHG process are imprinted in measurable photon statistics of the transmitted IR laser field, thus accessing HHG dynamics does not require measuring the XUV radiation. This is a unique advantage of our work since our proposed measurements, dealing with high-resolution spectroscopy in XUV and attosecond science, can be performed in open air without the need for specialized optics/diagnostics required for the characterization of the XUV radiation. Additionally, it has been found that the interaction of strong laser fields with gas phase media leads to the production of non-classical high photon number light states.

## Full-quantum theoretical description of light-electron interaction

The non-relativistic TDSE of an electron interacting with a single-mode long-wavelength linearly-polarized quantized light field of frequency *ω* reads (in atomic units)


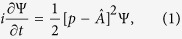


where *p* and 
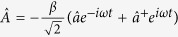
 are the electron momentum and vector potential scalar operators along the polarization direction. The creation and annihilation operators are 
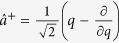
 and 
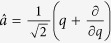
, respectively, *q* is the in-phase quadrature of the field^9^,^10^, and 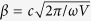 is a constant determined by the quantization volume *V*, frequency *ω*, and light velocity *c*. The detailed derivation of the analytical solution of Eq. (1), termed quantum-optical Volkov wavefunction, will be given elsewhere. Here we provide the result, the validity of which can be checked by direct substitution into Eq. (1). Based on this, we then analyze its fundamental features and their consequences for the HHG process. The closed-form solution of Eq. (1) reads:


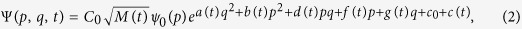


where the functions *a*(*t*), *b*(*t*), …., *g*(*t*), *M*(*t*) are given in terms of the parameters of Eq. (1) in the Methods Section. The solution includes an arbitrary initial electron distribution *ψ*_0_(*p*) and an arbitrary initial photon number *N*_0_ and the field phase *θ*. The wavefunction Ψ(*p, q, t*) provides the full quantum-optical description of the electron-light interaction. The term *d*(*t*)*pq* in the exponent renders the electronic and light degrees of freedom non-separable. In the high photon number limit where *N*_0_ → ∞, *β* → 0 (*V* → ∞), and 
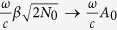
 (where *A*_0_ is the amplitude of the corresponding classically-described vector potential *A* = *A*_0_ cos(*ωt* + *θ*)), Eq. (2) is simplified (see Methods) to Ψ′(*p, q, t*) maintaining all the quantum-optical properties of Eq. (1).

A crucial property of the quantum-optical Volkov wave function is that the matrix elements of any *q*-independent operator 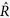 coincide with the matrix elements obtained from using the well known semi-classical electron Volkov wave functions *ψ*_*V*_ i.e.


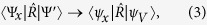


where Ψ_*x*_ is an arbitrarily chosen electron-light wave function and *ψ*_*x*_ is the corresponding state of the electron in case of classically-described electromagnetic field. Thus, while Ψ′(*p, q, t*) goes beyond the semi-classical approach to completely describe the quantized electron and light interaction, it naturally reproduces the classical Volkov states after integrating over *q*. This has profound consequences for the description of HHG, since the well known results of the semi-classical models^4^ can be retrieved, and more than that, utilized in our quantized-field approach. In the particular case of HHG, Ψ_*x*_ is the ground state of the system (*ψ*_*g*_*ψ*_*c*_), 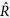 is the dipole moment 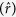, *ψ*_*x*_ is the ground state of the atom (*ψ*_*g*_) and *ψ*_*c*_ is the initial coherent light state. Detailed description of the above considerations can be found in the Methods Section.

The calculation of the dipole moment in the high photon number limit demonstrates that the behavior of the electron in a strong laser field can be accurately described by semiclassical theories with negligible quantum corrections. However, our full quantum-optical approach can provide information about the IR laser field states during the interaction, inaccessible by the semi-classical theories. This information can be experimentally extracted utilizing XUV/IR-correlation approaches and/or balanced homodyne detection techniques^11^,^12^,^13^ of the IR laser field transmitted from the harmonic generation medium.

## Quantum-optical description of the HHG process

Using the quantum-optical Volkov wave functions Ψ′ in high photon number limit, the time evolution of the HHG process is described by the following wave function


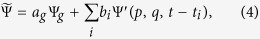


where Ψ_*g*_ = *ψ*_*g*_*ψ*_*c*_ is the initial state of the system, *ψ*_*g*_ is the ground state of the electron, *ψ*_*c*_ is the initial coherent light state and Ψ′(*p, q, t* − *t*_*i*_) are the continuum laser-electron states having different ionization times *t*_*i*_. In eq. 4 we assume that light-electron states (at any time t) can always be represented by a superposition of bound states (one bound state in our case) and continuum states. The amplitude of the bound state is *a*_*g*_(*t*), and the amplitudes of the continuum states are *b*_*i*_(*t*). In the same way as in the semi-classical theory^4^ we assume that *a*_*g*_(*t*) ≈ 1, while all 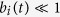. In addition, we choose (as initial condition) that before the ionization *a*_*g*_(*t*_0_) = 1 (where *t*_0_ < *t*_*i*_), so *b*_*i*_(*t*) = 0 for *t* < *t*_*i*_, and *b*_*i*_(*t*) = *b*_*i*_ for *t* > *t*_*i*_. The complex amplitudes *a*_*g*_ and *b*_*i*_ satisfy the normalization condition 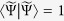. In this case, the time dependent dipole moment is 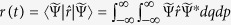. As in the semi-classial theory^4^, we neglect ground-ground 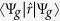 and continuum-continuum 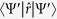 transitions, and consider only ground-to-continuum (and continuum-to-ground) transitions given by the matrix elements 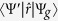 (and 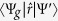).

Integrating 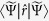 over *q*, using Eq. (4) and integrating over *p*, we retrieve that 
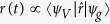
 which coincides with the expression given by the semi-classical theories. Thus, all the semi-classical results^4^,^14^,^15^,^16^, in particular the short (S) and long (L) electron paths with electronic Volkov wave functions 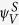 and 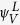 respectively, can be consistently used in the present approach. In a similar way, the same results can be obtained using the IR wave functions *ψ*_*l*_ (see Method section).

**Figure 1:**
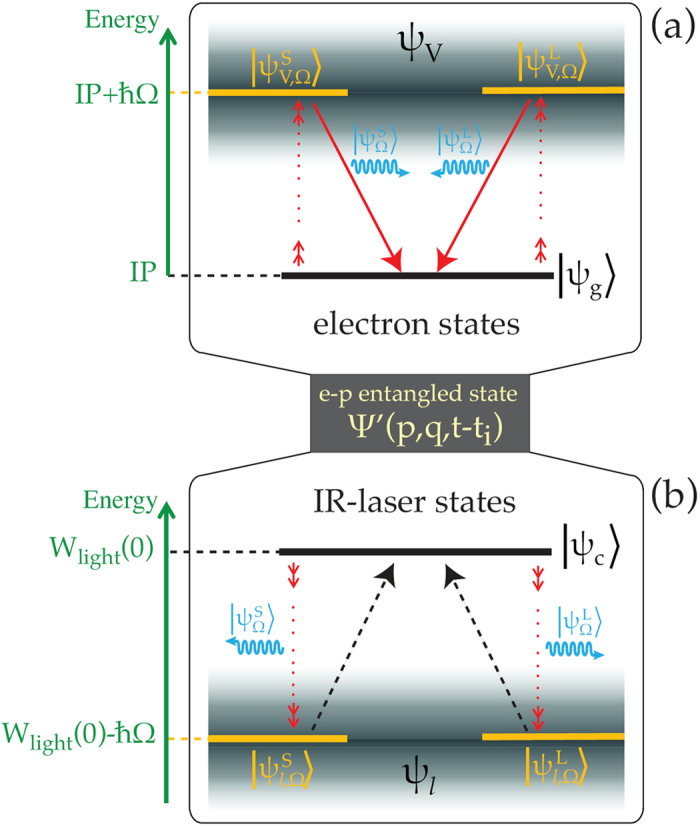
Excitation scheme for the quantum-optical description of the HHG process. (**a**) A schematic representation of the electron states in case of integrating over laser-state parameter *q*. (**b**) A schematic representation the laser field states in case of integrating over electron momentum *p*.

A scheme which can describe the HHG process in the context of the present model is shown in Fig. 1. Although the quantization of the harmonic field is not required for this work and thus was not considered in the previous formalism, harmonic photons are included in Fig. 1 for a complete understanding of the process. Figure 1a shows the electron states in case of integrating over *q* and Fig. 1b shows the field states in case of integrating over *p*. The horizontal black lines in Fig. 1a,b are the initial states of the electron |*ψ*_*g*_〉 and IR-laser field |*ψ*_*c*_〉 with energy *IP* < 0 and *W*_light_(0), respectively. At *t* > 0 the system is excited (small red arrows) in an infinite number of entangled laser-electron 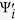 states (gray area), resulting in a reduction of the average laser energy (small downwards red arrows in Fig. 1b) and the enhancement of the average electron energy (small upwards red arrows in Fig. 1a). The Ω-frequency emission is taking place by constructive interference of *ψ*_*V*_ states and recombination to the ground state (downwards red arrows in Fig. 1a). In this case the final laser energy remains shifted by *ħ*Ω compared to the initial energy *W*_light_(0). When the *ψ*_*V*_ states interfere destructively the probability of Ω-frequency emission is reduced and the average laser energy returns to the initial value (black dashed arrows in Fig. 1b). Among the infinite 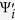 states, two are the dominant surviving the superposition, corresponding to the S and L electron paths, described by 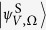 and 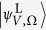, respectively. Correlated to them are the IR-laser states 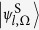 and 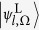 (where 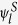 and 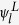 are the IR wave functions correspond to the S and L electron paths, respectively), as well as the Ω-frequency states 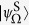 and 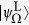, respectively. This is consistent with the interpretation of recent experimental data^17^. It is thus evident that by measuring quantum optical properties of the IR-light we can access the full quantum dynamics of the HHG process. Such properties, in particular photon statistics to be discussed next, are not accessible by semi-classical models^1^,^2^,^3^,^4^.

## Counting IR photons in HHG

**Figure 2:**
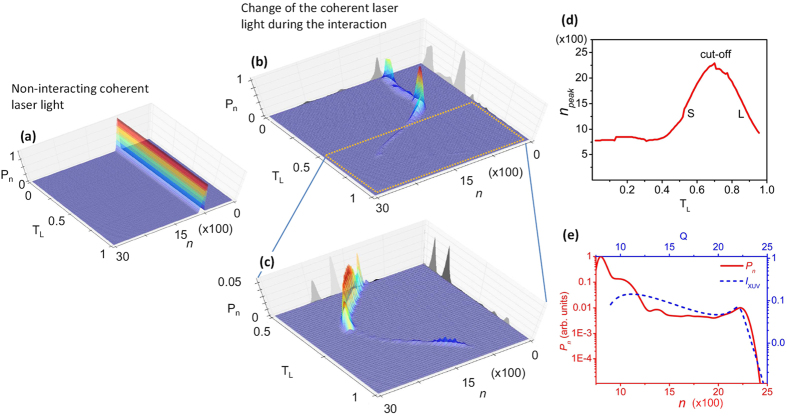
Probability distribution of the IR photons during the HHG process. (**a**) Probability distribution of a non-interacting coherent IR laser state for *N*_0_ = 800. This is shown only for reasons of comparison with the interacting coherent laser states. (**b**) Time dependence of the IR probability distribution during the recollision process calculated for 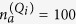 and *I*_*l*_ = 10^14^ *W*/*cm*^2^. In the calculation, the electron momentum, the ionization and the recombination times have been obtained by the 3-step semi-classical model. (**c**) Expanded plot of (**b**) in the time interval 0.5*T*_*L*_ < *t* < *T*_*L*_. (**d**) IR photon number absorbed by Xenon atoms during the recollision process. This has been obtained by the *n* = *n*_*peak*_ position of the peak of the distribution at each moment of time. (**e**) Overall IR photon number absorbed by the atoms during the recollision (red solid line). This has been obtained after integration over the cycle of the IR field. The XUV spectrum shown in blue dashed line, obtained using the semi-classical 3-step model.

The probability for measuring *n* photons in a non-interacting coherent light state is given by *P*_*n*_ = |*K*_*n*_|^2^, where *K*_*n*_ is a probability amplitude appearing in the expansion 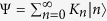, in terms of photon-number (Fock) states. In this expression, *K*_*n*_ is time-independent, since, as well known^18^, the photon probability distribution in a coherent state is constant within the cycle of the light field (Fig. 2a). When the coherent light state is interacting with a single atom towards the generation of XUV radiation, the probability distribution becomes time dependent, since Ψ′(*p, q, t* − *t*_*i*_) is changing at each moment of time within the cycle of the laser field due the interaction with the ionized electron. In this case, the probability distribution is given by 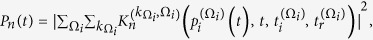 where 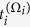 and 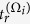 are the ionization and recombination times of the corresponding electron paths 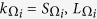 with momentum 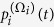 which lead to the emission of XUV radiation with frequency Ω_*i*_ (see Methods Section). The parameters 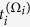, 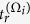 and 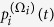 are obtained using the 3-step semi-classical model^4^.

In reality, an intense Ti:S femtoseond (fs) laser pulse with ~10^17^ photons/pulse (which corresponds to *N*_0_ ~ 10^12^ photons/mode for a laser system based on a 100 MHz oscillator which delivers pulses of ~30 fs duration), interacts with gas-phase medium towards the emission of XUV radiation. In this case, where 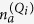 atoms coherently emit XUV radiation with frequencies proportional (*Q*_*i*_ = Ω_*i*_/*ω*) to the frequency of the IR laser, the interaction is imprinted in the photon number 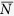 of the IR field as 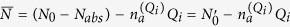, reflecting energy conservation (*N*_*abs*_ is the number of those photons that do not lead to XUV emission during the recollision process). Since the signal of interest, 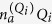, is superimposed on a large background 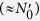 which for reasons of simplicity is set 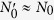, a XUV/IR correlation approach and/or a balanced interferometer^11^,^12^,^13^ is required in order to subtract the IR photon number *N*_0_ from 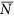 and thus measure 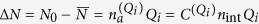. The number of atoms interacting with the laser field is *n*_int_ and 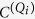 is the conversion efficiency of a single-XUV-mode, which depends on the gas density in the interaction region. A rough estimation of Δ*N* can be obtained by taking into account the typical harmonic conversion efficiency and gas density values used in a harmonic generation experiment. The propagation effects of the IR beam in the medium have not been considered, as the precise calculation of Δ*N* is out of the scope of the present work. Taking into account that for gas densities ~10^18^ atoms/cm^3^ the conversion efficiency is ~10^−4^ (for Argon, Krypton, Xenon in the 25-eV photon energy range)^19^,^20^,^21^,^22^,^23^, it can be estimated that Δ*N* ranges from ~0 (for zero gas density) up to ~10^9^ photons/mode (for gas density ~10^18^ atoms/cm^3^). Although the study is valid for all noble gases, in the following we will describe the HHG process considering Xenon atoms interacting with a coherent IR laser field in case of low 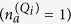, intermediate 
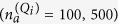
 and high 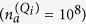 number of emitting atoms.

For a single recollision, the dependence of *P*_*n*_(*t*) on time during the process is shown in Fig. 2b,c for 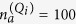. It is seen that in the time interval 0 < *t* < *t*_*i*_ ≈ 300 asec where the ionization is taking place, the peak of the probability distribution is located at *n* = *n*_*peak*_ ≈ 800. Since the ionization of one Xenon atom requires the absorption of *n* ≈ 8 IR photons, this value corresponds to the energy absorbed by 100 Xenon atoms. For *t* > *t*_*i*_ the variation of the IR photon number *n* with time reflects the energy exchange between the IR laser field and the free electron. The peak of the probability distribution during the recollision is located at *n* = *n*_*peak*_ = Ω_*i*_/*ω*, 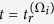 (Fig. 2d). This is due to the energy absorbed by Xenon atoms during the recollision process towards the emission of XUV radiation with frequency Ω_*i*_ at the moment of recombination 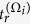. Importantly, in Fig. 2d we demonstrate that the absorbed IR photon number reveals the fundamental properties of the three-step semi-classical model: S and L paths lead to the emission of the same XUV frequency and degenerate to a single path in the cut-off region. Furthermore, as shown in Fig. 2e, the overall IR photon number distribution (red solid line) reproduces the well-known XUV spectrum resulting from the semi-classical three-step model (blue dashed line), including the plateau and cut-off regions. Thus, we demonstrated that all known features of the semiclassical three-step model are imprinted in IR photon statistics.

**Figure 3:**
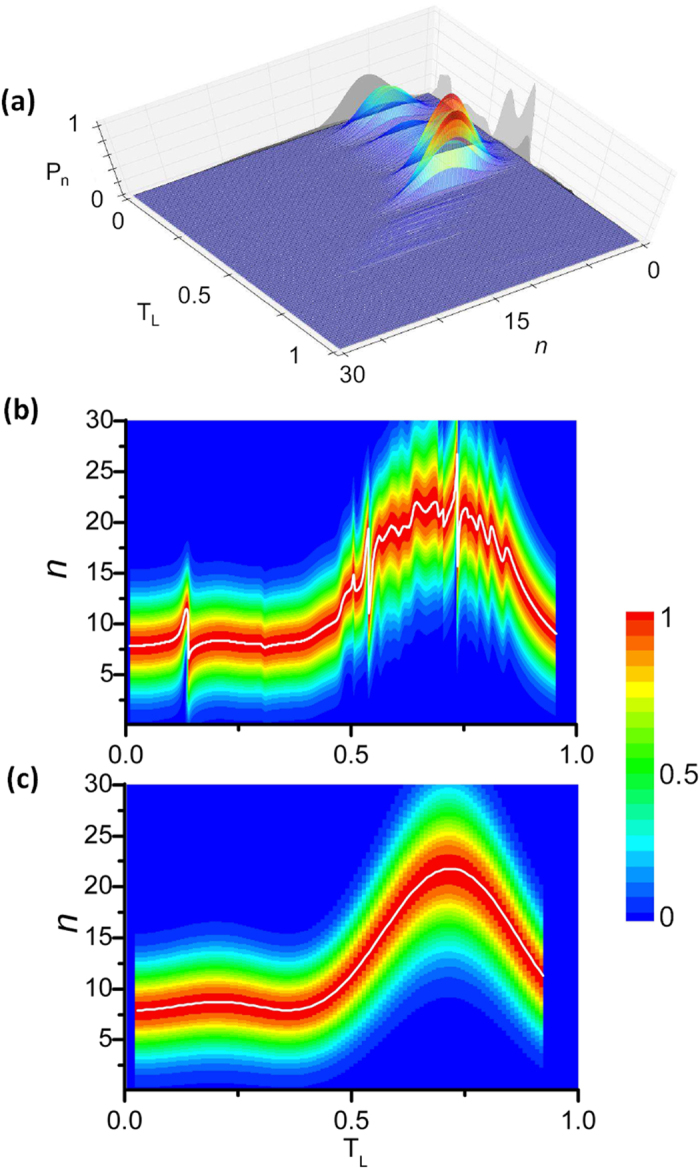
Generation of light states with non-Gaussian photon distribution. (**a**) Time dependence of the IR probability distribution during the recollision process calculated for 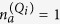 and *I*_*l*_ = 10^14^ *W*/*cm*^2^. In the calculation, the electron momentum, the ionization and the recombination times have been obtained by the 3-step semi-classical model. (**b**) Contour plot of the normalized probability distribution of (**a**). (**c**) Contour plot of the normalized probability distribution which follows the Gaussian photon statistics. This has been calculated using a single electron path which contributes to the emission of a monochromatic XUV radiation with frequency *Q*_*i*_ = *ω*/11. It is evident, that in case of reducing the number of emitting atoms form 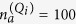 (Fig. 2b,c) to 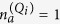 the width of the probability distribution is increasing.

We will now explore the new phenomena and potential metrological applications one can address utilizing IR photon statistics. To this end, we first elaborate on the atom-number dependence of *P*_*n*_. While the number of IR photons absorbed by the system is proportional to 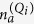, the width 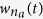 of the probability distribution is determined by Gaussian statistics, 
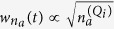
 (Fig. 3a). However, the distribution is departing from the Gaussian statistics during the recollision process. This is clearly shown in Fig. 3b which depicts in contour plot the normalized probability distribution of Fig. 3a. For reasons of comparison, a Gaussian distribution is shown in Fig. 3c. The distortion of the probability distribution in Fig. 3b, more pronounced in the time interval 0.5*T*_*L*_ < *t* < *T*_*L*_, is associated with energy/phase dispersion of the interfering electron wave packets in the continuum, alluding to the possibilities of producing non-classical light-states.

**Figure 4:**
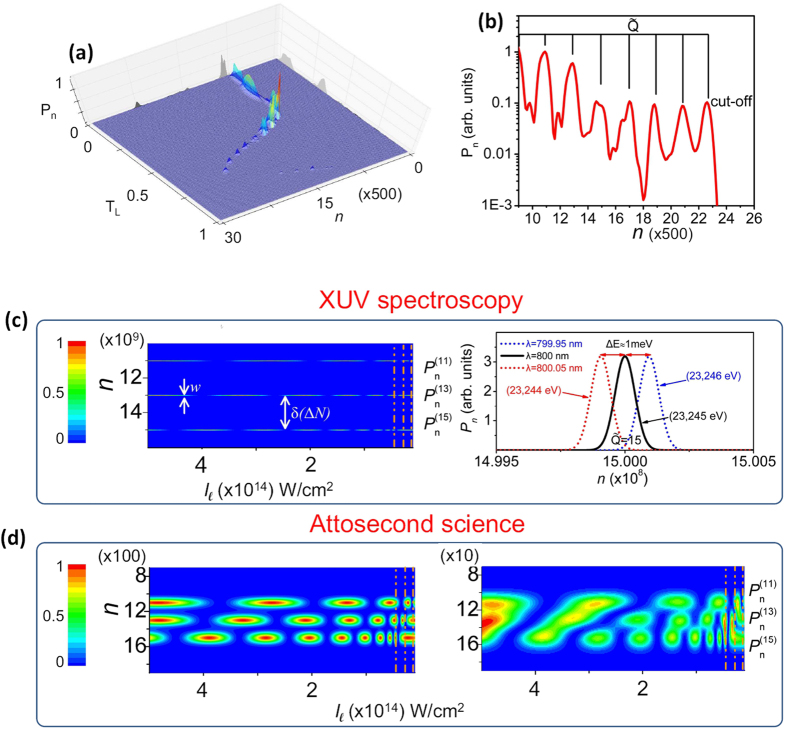
High resolution spectroscopy in XUV and attosecond science using IR photon statistics. (**a**) Probability distribution for multi-cycle laser interaction calculated for 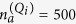 and *I*_*l*_ = 10^14^ *W*/*cm*^2^. For this graph three laser cycles have been considered. (**b**) “IR photon statistics spectrum” obtained by time integrating the (**a**). (**c**) (left panel) Dependence of *P*_*n*_ on the laser intensity *I*_*l*_ (∝*N*_0_) and on photon number *n* for 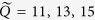 and 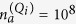. The right panel shows the probability distribution around 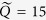 in case of recording the 799.95 nm, 800.00 nm and 800.05 nm IR modes of the laser pulse after passing through the gas medium. (**d**) Dependence of *P*_*n*_ on the laser intensity and on photon number *n* for 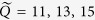 and for 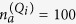 (left panel) and 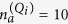 (right panel). The dashed vertical lines depict the cut-off positions of the harmonics. In these plots 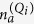 were taken independent of *I*_*l*_.

For multi-cycle laser field, the process is repeated every half-cycle of the laser period. In this case the probability distribution consists of a series of well confined peaks (Fig. 4a,b) appearing at positions 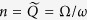 and reflects the formation of well confined high order harmonics 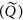.

Additionally, the atom-number dependence of the IR photon distribution in the HHG process provides significant advantages for high resolution spectroscopy in XUV and attosecond science. In Fig. 4c (left panel) we show the dependence of the *P*_*n*_ on the intensity of the laser field (*I*_*l*_ = *ε*_0_|*E*_0_|^2^/2 ∝ *N*_0_) and *n* for 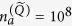 (for simplicity we consider only the case of 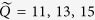). Indeed, the harmonic spectrum can be obtained from the maxima of *P*_*n*_ centered at 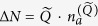. The spacing between the maxima 
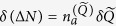
 with 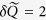 for consecutive harmonics, and the width 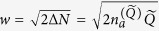 depend on 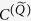 and *n*_int_. The resolving spectral power 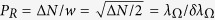 increases with *n*_int_ and for values of Δ*N* ~ 10^9^ photons, *P*_*R*_ can reach the values of ~10^4^–10^5^ in the spectral range of 25 eV, which competes with state-of-the-art XUV spectrometers. This is shown in Fig. 4c (right panel) where the probability distribution around 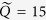 has been calculated in case of recording the 799.95 nm, 800.00 nm and 800.05 nm IR modes of a Ti:S laser pulse. This measurement can be performed by collecting the photons of the IR modes of the spectrally resolved multi-color IR pulse. This can be done by means of an IR diffraction grating placed after the harmonic generation medium. This figure also depicts the broadening effects introduced in a measured distribution by the bandwith of the driving IR pulse in case of collecting more than one modes of the multi-mode laser pulse.

When Δ*N* is reduced, the probability distribution is getting broader (Fig. 4d, left panel), while at the point where the probability distribution between the consecutive harmonics overlaps, an interference pattern associated with the relative phase between the consecutive harmonics appears in Fig. 4d (right panel). Additionally, the modulation of *P*_*n*_ with the intensity of the laser field (clearly shown in the left panels of Fig. 4c,d) reflects the effect of the S and L path interferences in the context of Fig. 1, i.e. the maxima (minima) of *P*_*n*_ versus *N*_0_ correspond to those IR-laser intensities *N*_0_, for which Ψ_*V*_ interferes destructively (constructively). These observations can be used for attosecond science and metrology, to be explored in detail elsewhere. Since the photon statistics measurements are sensitive to shot-to-shot fluctuations of the IR intensity, stable laser systems or IR energy tagging approaches are required in order to be able to record an “IR photon statistics spectrum”. Additionally, in order to avoid the influence of the laser intensity variation along the propagation axis in the harmonic generation medium, a gas medium with length much smaller compared to the confocal parameter of the laser beam is required. Any influence of the intensity variation along the beam profile at the focus can be minimized (in case that is needed) using spatial filtering approaches where the IR photons of the specific area on the focal spot diameter can be collected.

## Conclusions

Concluding, we have developed a quantized-field approach which describes the strong-field light-electron interactions using a quantized coherent laser state with arbitrary photon number. The description is based on the quantized-Volkov light-electron wave function resulting from the closed-form solution of TDSE. The obtained wave function provides information about the quantum optical features of the interaction, which are not accessible by the semi-classical approaches used so far in strong-field physics and attosecond science. The approach has been used for the description of HHG in gases. We have found that the quantum optical features of the HHG can be unraveled by measuring the photon statistics of the IR laser beam transmitted from the gas medium without the need of measuring the XUV radiation. This is a unique advantage of the work since our proposed measurements, dealing with high-resolution spectroscopy in XUV and attosecond science, can be performed without the need for specialized XUV equipment (gratings, mirrors, high vacuum conditions etc.). Additionally, we have found that the HHG process in gases can lead to non-classical IR light states. In general, this work establishes a promising connection of strong-field physics with quantum optics.

## Methods

### On the closed-form solution of TDSE

In order to obtain a closed-form solution of Eq. (1) of the main text of the manuscript, we consider as an initial state, a state where the electron is decoupled from the light i.e. 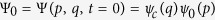 to be a separable product of a coherent state of light 
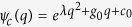
 and an arbitrary field-independent electron state *ψ*_0_(*p*) in momentum representation, where 
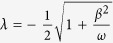
 is the parameter which introduces the light dispersion due to the presence of the electron^24^,^25^, 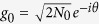 carries the information about the phase of the light *θ, N*_0_ is the average photon number of the initial (*t* = 0) coherent light state, and *c*_0_ is a normalizing constant.

The parameters appearing in Eq. (2) of the main text of the manuscript are


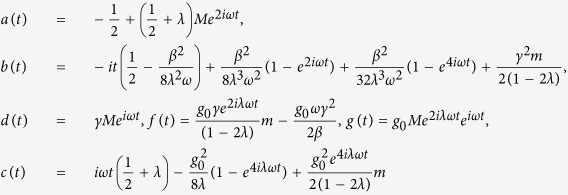


where 
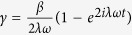
, 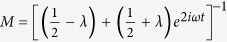, *m* = 1 − *Me*^2*iωt*^, 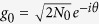, 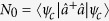 and *C*_0_ is normalization constant. From the general solution of Eq. (2) we can recover energy conservation, i.e. the instantaneous interaction energy of the electron is given by *W*_e,int_(*t*) = *W*_*e*_(*t*) − *W*_*e*_(0) = *W*_light_(0) − *W*_light_(*t*), where *W*_*e*_(0) is the initial kinetic energy of the electron, 
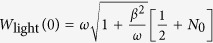
 is the initial energy of the light field, 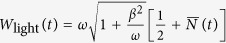 is the field energy at any moment of time and 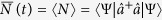. In the high photon number limit the *q*-dependent part of the total wave function becomes exponentially small everywhere except the region around 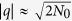. Thus, Eq. (2) of the main text of the manuscript leads to


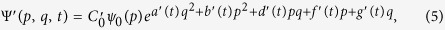


where now the parameters in the exponent are


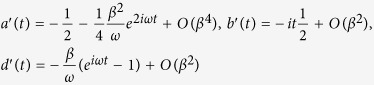



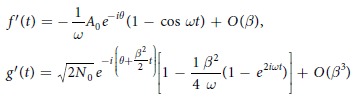


and 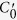 is normalization constant.

### On the validity of Eq. (3)

Equation (3) of the main text of the manuscript can be proved in the following way (since the origin of time *t* can be arbitrary chosen, for simplicity and without loss of generality we set *θ* = 0). For an arbitrary Ψ_*x*_(*p, q, t*) and 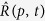, in the high photon number limit (where *N*_0_ → ∞, 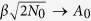 and 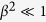) the matrix element 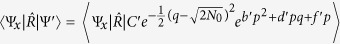 (where 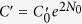) with the exponent 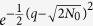 to be proportional to 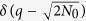. The integration over *q* i.e. 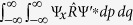, leads to 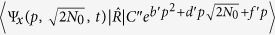 = 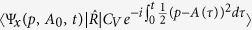 = 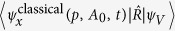 = 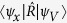, where 
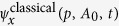
 is the arbitrary state of system with classically described electromagnetic field and 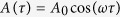.

### On the description of HHG using the IR wave functions

The results obtained by the semi-classical theories regarding HHG can be also obtained by integrating 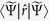 over *p*, using Eq. (3) and integrating over *q*. In this case the dipole moment 
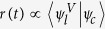
 is expressed in terms of the corresponding to the Volkov-electron states IR wave functions 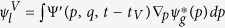 (where *ψ*_*g*_(*p*) is a ground state of the electron in momentum representation, *t*_*V*_ are the ionization times Volkov electron paths contribute to the harmonic generation). The IR wave functions which correspond to the S and L electron paths are 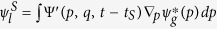 and 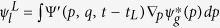, respectively, with *t*_*S*_ and *t*_*L*_ being the ionization times of the short and long electron paths.

### On the calculations of the IR probability distribution

The probability to measure *n* photons in a non-interacting light field state Ψ is *P*_*n*_ = |*K*_*n*_|^2^, where *K*_*n*_ is a probability amplitude appearing in the expansion 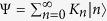, in terms of photon-number (Fock) states. In *q*-representation the Fock states are written as 
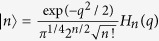
, where 
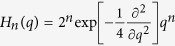
 are Hermite polynomials. For coherent light states^9^, the photon statistics are described by the Poisson distribution 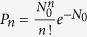, well-approximated by a Gaussian 
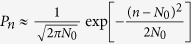
 when 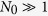. In case of HHG process, the probability distribution during the recollision process for a single path *i* of ionization time *t*_*i*_ and electron momentum *p*_*i*_(*t*) which contributes to the production of XUV radiation with frequency the harmonic Ω_*i*_ is given by 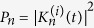, where 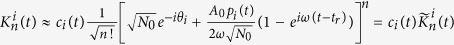 is determined through the expansion 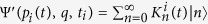. *c*_*i*_(*t*) are *n*-independent complex numbers proportional to the *q*-independent part of the Ψ′ and *θ*_*i*_ = *ω*(*t* − *t*_*i*_) is the phase of the laser field at the moment of ionization. In the high photon number limit, 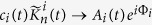 (where *A*_*i*_(*t*) is real), and the probability distribution reads 
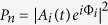
, with


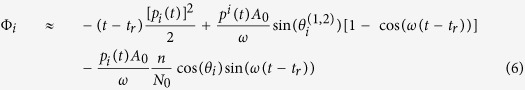


where 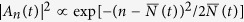 and 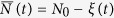 is the average number of photons during the recollision, with 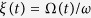 and Ω(*t*) = ((*p*^2^(*t*)/2) − *IP*). When multiple paths contribute to the emission of multiple harmonics, 
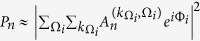
, where 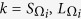 denotes the electron paths contribute to the emission of the Ω_*i*_ frequency. Since the probability distribution during the recollision is located at (*n* = *n*_*peak*_ = Ω_*i*_/*ω*, 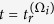) the above expression of *P*_*n*_ and Φ_*i*_ can be further simplified by omiting the time *t*. This is very useful for calculating the dependence of *P*_*n*_ on the intensity of the laser field as is shown in Fig. 4.

## Additional Information

**How to cite this article**: Gonoskov, I. A. *et al*. Quantum optical signatures in strong-field laser physics: Infrared photon counting in high-order-harmonic generation. *Sci. Rep.* **6**, 32821; doi: 10.1038/srep32821 (2016).
